# Influences of Different Land Use Spatial Control Schemes on Farmland Conversion and Urban Development

**DOI:** 10.1371/journal.pone.0125008

**Published:** 2015-04-27

**Authors:** Min Zhou, Shukui Tan, Lu Zhang

**Affiliations:** 1 Non-traditional Security Center of Huazhong University of Science and Technology, Wuhan, China 430074; 2 College of Public Administration, Huazhong University of Science and Technology, Wuhan, China 430074; DOE Pacific Northwest National Laboratory, UNITED STATES

## Abstract

Land use planning is always officially implemented as an effective tool to control urban development and protect farmland. However, its impact on land use change remains untested in China. Using a case study of Hang-Jia-Hu region, the main objective of this paper was to investigate the influence of different land use spatial control schemes on farmland conversion and urban development. Comparisons of farmland conversion and urban development patterns between the urban planning area and the non-urban planning area were characterized by using remote sensing, geographical information systems, and landscape metrics. Results indicated that farmland conversion in the non-urban planning area was more intensive than that in the urban planning area, and that farmland patterns was more fragmented in the non-urban planning area. Built-up land patterns in the non-urban planning area showed a trend of aggregation, while those in the urban planning area had a dual trend of fragmentation and aggregation. Existing built-up areas had less influence on built-up land sprawl in the non-urban planning area than that in the urban planning area. Built-up land sprawl in the form of continuous development in the urban planning area led to farmland conversion; and in the non-urban planning area, built-up land sprawl in the form of leapfrogging development resulted in farmland areal declines and fragmentation. We argued that it is a basic requirement to integrate land use plans in urban and non-urban planning areas for land use planning and management.

## Introduction

Built-up land now covers about 400,000 km^2^ of the global terrestrial surface, and the number is estimated to reach 700,000 km^2^ by 2030 [1]. Built-up land area in developing countries is expected to triple from 200,000 km^2^ to 600,000 km^2^, and that in developed countries is projected to increase from 200,000 km^2^ to 500,000 km^2^ [2]. The rapid urban development worldwide, as a consequence not just of population boom but also of new urban lifestyles that desire more space [3], involves two processes: sprawl and fragmentation. Sprawl refers to the physical growth of built-up land area, conversing from non-urban (open or natural) land to built-up land [4]; and fragmentation denotes the morphological changes of built-up land patches [5]. These two processes, evidenced by many cases, have raised numerous ecological problems [6,7,8]. A typical ecological consequence is the depletion of farmland. Scholars have report the occupation of prime farmland due to sprawl in many places around the world [9,10,11,12,13]. The sharp conflict between urban development and farmland depletion poses great challenges to global and regional sustainable development, given that it is tightly related to food security [14,15].

Land use planning is always officially implemented as an effective tool to restrain sprawl and protect farmland [16]. In China, land use planning did exert some positive effects on farmland protection [17,18]; however, massive farmland is still converted to non-agricultural uses [14,19,20,21]. Two major factors contributed to this phenomenon: for one thing, land use planning has not been adjusted timely in accordance with socioeconomic development and it is not complied strictly in practice [18]. For another, within the context of China’s land use planning system, land use is managed by different levels of government and agencies [18]. Consequently, land use control schemes are divided into two spatial domains (the urban planning area and the non-urban planning area). These two planning areas are differed with intensity and flexibility in land development, which should lead to discrepancy of land use patterns. However, the influence of different land use spatial control schemes on farmland conversion and urban development is untested in China.

It requires a full account of land use characteristics to quantify the impact of land use planning, which divides the land use control schemes into two spatial domains. Land cover information can be detected by remote sensing (RS) [22,23,24] and the sprawl process can be analyzed by geographical information systems (GIS) [7]. Landscape metrics provide critical tools in analyzing, describing, and modeling the structural patterns of land use [25,26], and therefore can be applied to measure the fragmentation process [5]. This paper applied these tools into the case of Hang-Jia-Hu region in Chinese eastern coast. Our objectives are to: (1) compare the spatiotemporal patterns of urban development and farmland conversion in different spatial planning areas; (2) analyze how farmland conversion is related to urban development and land use planning; and (3) discuss the impacts of spatial planning on land use change.

## Background

### Spatial Planning

Spatial planning, allocating different land uses to specific units by weighing the trade-offs among conflicting, has been implemented in many countries and regions, including the Italy, Spain, UK, Germany, Netherland, USA, Irish, China, and so on [27,28,29,30]. Within the context of spatial planning systems, it involves different levels of management main bodies, ranging from national to regional and local institutions. Besides, the land use control schemes are divided into different spatial domains. For example, several countries divide the spatial domains into urban, exurban, and rural planning areas, while the watershed is used as the unit in spatial planning for water quality management in the European Unions.

Coming into effect from April 1st 1990, China's “Urban Planning Law” divides the land planning into two spatial domains: the urban planning area and the non-urban planning area. Article III states that: the urban planning area mentioned in this Law refers to the urban areas, suburban areas and urban administrative areas which need control due to urban construction and development. The main objective of delineating the urban planning area is to control land use of urban construction and to ensure the progressive realization of the overall urban master plan.

Urban planning area generally consists of three categories: (1) the urban built-up area. The main task of land use management in this context is to rationalize and control new construction and alteration of urban facilities, and to adjust and develop existing land reasonably. (2) the spatial scope of land that urban areas or cities need for long-term development. This kind of urban planning area includes independent land outside built-up areas, water source and protection land, airport and its control areas, radio stations, scenic spots, historical and cultural heritage areas and so on. The main task of land use management in this context is to ensure that development and construction of land and facilities is done in an orderly manner. (3) suburbs. Land use management in this context is closely linked with urban development. Therefore strict control is placed on land use in urban areas and rural settlements within the region.

Though the “Urban Planning Law” marks the progress of China’s society and serves as recognition of the spatial planning practice, it raises many questions in practice: (1) Land management is determined at different levels of government and administrative units. The administrative main body of China’s spatial planning system consists of three ones, namely, “land use planning” led by the Ministry of Land Resources, “regional planning” led by the National Development and Reform Commission and “urban planning” led by the Ministry of Construction. The three administrative departments make and implement land use planning within their respective administrative systems framework. Therefore, the land use patterns should present different characteristics under different land use planning domains. (2) During the process of urbanization, in many places of China, especially in the developed coastal regions, cities and surrounding towns and villages have sprawled fast. This kind of settlement sprawl or contiguous development is often beyond the urban planning area. If still using the planning model, it is impossible to fully tackle with this problem. (3) Inadequate attention is given to the non-urban planning area. Many rural places and villages in the land-use plan outside the urban planning area is a blank. But the fact is that those village settlements in the region may already be integrated into the urban build-up area, indicating deficiency of the land-use planning system.

### Study Area

**Fig 1:**
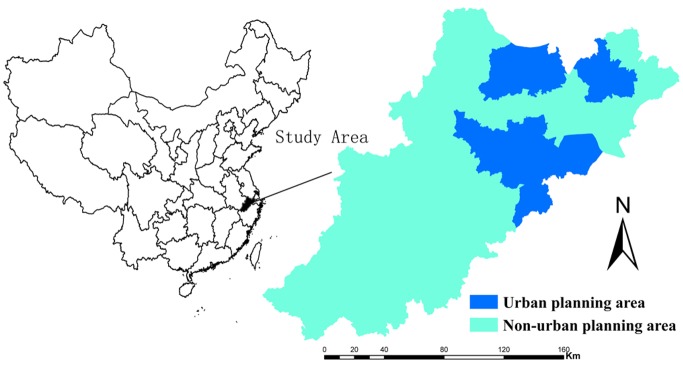
Location and spatial planning domains of Hang-Jia-Hu region, China.

Hang-Jia-Hu region is constituted by three cities (Hangzhou, Jiaxing and Huzhou) in Zhejiang province and has a population of approximately 14 million. Lying on the Grand Canal of China, it borders Lake Tai to the north, Shanghai to the northeast, and Jiangsu Province to the northwest (Fig 1). This region enjoys a warm temperate, subtropical monsoon climate, with annual temperature averaging 17.5°C, rainfall averaging 1139 mm and sunshine hours amounting to 1762 h. Benefiting from these natural conditions, yields are high and agricultural cultivation has a long history. This region has been undergoing rapid socioeconomic development since China’s market transition in 1994. Its total gross domestic product was tripled from 1994 and 2003, growing from 125 billion dollars in 1994 to 467 billion dollars in 2003 [31]. The accelerating socioeconomic development promoted urban development and the subsequent farmland conversion. We can therefore refer to the Hang-Jia-Hu region as a typical case for investigating the impacts of spatial planning on urban development and farmland conversion.

## Materials and Method

### Data source and processing

**Fig 2:**
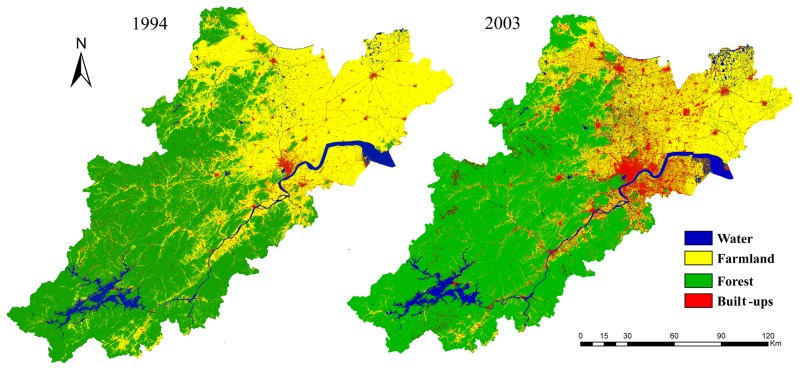
Land cover patterns of Hang-Jia-Hu region in 1994 and 2003 (Data source: [31]).

Land cover information (built-up land and farmland) was obtained from [31]. According to [31], land cover information in 1994 and 2003 was interpreted from Landsat TM images and Landsat ETM+ images, respectively. After geo-registration, these images were subjected to multiple endmember spectral mixture analysis [32], considering the fragmented land use patterns in the study area. In particular, farmland in this study refers to the land used to grow crops [31]. The interpreted land cover maps (Fig 2) were integrated into a GIS platform and overlaid to determine the patterns of farmland conversion and built-up land sprawl between 1994 and 2003.

The conversion rate for farmland was calculated according the following formula.

$$R={\left[1-\frac{S_{1}-S_{2}}{S_{1}}\right]}^{\frac{1}{n}}-1$$ (1)

where R is the rate of conversion process, S_1_ the area of farmland at the date t_1_; S_2_ the area of farmland at the date t_2_; and n the difference of years between the two dates (10 years between 1994 and 2003 in this case study).

Moran’s I was calculated to characterize the spatial dependence of built-up land sprawl over time. The theoretical value of Moran’s I ranges from -1 to +1 (Moran, 1950). Higher value of Moran’s I from 0 to +1 denotes higher positive spatial dependence, which implies that existing built-up land has greater influence on sprawl. Lower value of Moran’s I between 0 and -1 denotes higher negative spatial dependence, which suggests that built-up land patches become more isolated in space. Calculation of Moran’s I was performed using ArcGIS 10.2 using the distance weight matrix.

### Landscape metric analysis

Scholars have proposed various landscape metrics to describe fragmentation; however, applying a set of metrics is superior to employing all the metrics for sake of the redundancy problem [33]. A set of class level metrics were first selected after literature review, and then redundancy was reduced by varimax rotation principal component analysis [7, 34]. The final selected metrics included number of patches (NP), patch density (PD), largest patch index (LPI), landscape shape index (SHAPE), patch cohesion index (COHESION), aggregation Index (AI) and splitting Index (SPLIT). It should be mentioned that fragmentation here has two different meanings. For built-up land fragmentation, it denotes the fragmentation process involved in urban development. Two types of built-up land sprawl exists: the continuous development (new development occurs adjacent to existing built-up land area), and leapfrogging development (new development rises from the existing built-up land area discontinuously) [5]. The continuous development, when it does not follow the main development axis, would increase the complexity of urban form. The leapfrogging development always destroys the integrity of natural landscapes, since the scattering patterns of built-up land complicate its morphology [34]. Consequently, the fragmentation process of urban development poses great threat to natural resources protection [34]. For farmland fragmentation, it refers to the process that entities supposed to be cohesive for optimally functioning are segregated in space [35]. Farmland fragmentation, which can impair the ecological functions of agricultural systems and lower their production efficiency, is not desirable in land management [36]. All the metrics were calculated using FRAGSTATS 3.3 [25], which is the most popular software to calculate landscape metrics.

## Results

### Spatial patterns of urban development

From 1994 to 2003, Hang-Jia-Hu region experienced obvious built-up land sprawl. Built-up land area increased from 26376 ha to 121665 ha in the urban planning area, and that in the non-urban planning area increased from 43532 ha to 105341 ha. Patterns of built-up land in Hang-Jia-Hu region showed a dual trend of fragmentation and aggregation. For one thing, the fragmentation was evidenced by the significant increases of NP, SHAPE, and SPLIT for built-up land (Table 1). For another, AI had been keeping increasing during the ten years. From the rising trend of COHESION, it can be clearly seen that built-up land patterns in 2003 turned more aggregated compared to those in 1994. Specifically, the fragmentation was more obvious in the non-urban planning area, with an increase in SPLIT and significant decline in COHESION (Table 1).

**Table 1:** Landscape metrics for built-up land fragmentation in Hang-Jia-Hu region in 1994 and 2003.

Landscape metrics	1994			2003		
	Whole Region	Urban Planning Area	Non-urban Planning Area	Whole Region	Urban Planning Area	Non-urban Planning Area
NP	99293	32171	67208	146608	53998	92967
PD	166.8	123.2	201.2	65.9	44.4	92.2
LPI	7.9	18.0	1.23	13.1	23.3	1.67
SHPAE	6.0	10.5	2.4	19.0	29.7	4.7
COHESION	93.0	96.7	81.3	98.5	99.3	93.3
AI	57.5	63.2	53.0	72.1	75.9	67.6
SPLIT	146.5	29.0	1611.8	42.0	13.4	480.9

Abbreviations: number of patches (NP), patch density (PD), largest patch index (LPI), landscape shape index (SHAPE), patch cohesion index (COHESION), aggregation Index (AI) and splitting Index (SPLIT).

The Moran’s I value of built-up land for the whole region was 0.31 in 1994, and rose up to 0.52 in 2004, denoting an increased dependence of built-up land. The spatial autocorrelation of built-up land in the urban planning was greater than that in the non-urban planning area (Table 2). Higher value of Moran’s I denotes that built-up land sprawl occurs along the boundaries of existing urban areas. These results implied that the continuous development was smaller than the leapfrogging development in the non-urban planning area, and the existing built-up areas had a less influence on built-up land sprawl in the non-urban planning area than that in the urban planning area.

**Table 2:** Moran’s I values for built-up land patterns in Hang-Jia-Hu region in 1994 and 2003.

	1994		2003	
	Moran’s I	P	Moran’s I	P
Whole Region	0.31	0.04	0.52	0.02
Urban Planning Area	0.55	0.02	0.68	0.01
Non-urban Planning Area	0.23	0.04	0.45	0.02

### Spatial patterns of farmland conversion

Fragmentation, evidenced by the 26.9% increase of PD, was the most obvious characteristics of farmland pattern changes (Table 3). The continuous decline of AI and LPI (Table 3) also indicated the fragmentation of farmland. In the urban planning area, SHAPE increased from 43.0 to 68.1, and that in the non-urban planning area amounted to 87.4 in 2003. The rapid increase of LSI denoted the farmland fragmentation in both planning areas. Nevertheless, the most interesting result, when we compared the farmland patterns in different spatial planning domains, was that NP for urban planning area showed a dramatic decline, while NP and LPI for the non-urban planning area increased slightly.

**Table 3:** Landscape metrics for farmland fragmentation in Hang-Jia-Hu region in 1994 and 2003.

Landscape metrics	1994			2003		
	Whole Region	Urban Planning Area	Non-urban Planning Area	Whole Region	Urban Planning Area	Non-urban Planning Area
NP	160543	18337	142594	146416	28070	118676
PD	13.9	3.9	20.7	17.0	8.4	22.6
LPI	75.7	27.7	34.9	71.3	25.4	48.4
SHPAE	128.8	43.0	57.4	165.4	68.1	87.4
COHESION	100	99.9	99.8	99.9	99.9	99.8
AI	90.8	94.9	88.0	86.0	87.8	84.8
SPLIT	1.7	4.7	5.3	2.0	5.8	3.6

Abbreviations: number of patches (NP), patch density (PD), largest patch index (LPI), landscape shape index (SHAPE), patch cohesion index (COHESION), aggregation Index (AI) and splitting Index (SPLIT).

### Impact of settlement sprawl on farmland conversion

**Fig 3:**
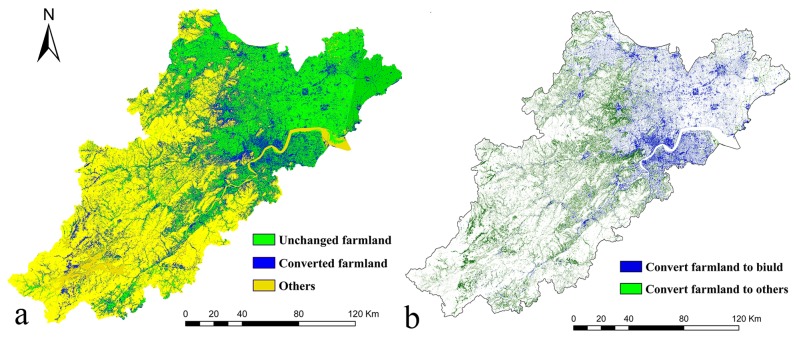
Spatial patterns of farmland conversion in Hang-Jia-Hu region in 1994 and 2003.

Fig 3 showed a clear pattern of farmland conversion to built-up land. Statistical results demonstrated that each spatial planning domain lost a comparable percentage of farmland converted to built-up land: urban planning area lost 74.0% at the annual rate of 16.8%, while non-urban planning area losing 32.5% of total farmland acreage at the annual rate of 21.0%. Analyzing the spatial relationships between the farmland conversion and built-up land sprawl, it was found that farmland in the urban planning area was mainly occupied by the continuous development, which spread progressively into adjacent farmland; and outside the urban planning area, most new development was in the form of leapfrogging, and scattered distributed in the farmland.

## Discussion and Conclusions

Spatial planning targeted at sustainable land use in urban areas, suburban areas and urban administrative areas, promoting built-up land construction and at the same time still protecting the natural environment [16,37]. Regarding the farmland protection in China, spatial planning is supposed to retain the fragmentation of built-up land area during sprawl, which can directly leads to the farmland fragmentation [36]. As shown by our study, however, the spatial planning has generated unintended consequences in the study area, since fragmentation of built-up area has been intensified in the urban planning area. This has clearly had a major impact on farmland patterns, generating more fragmented and isolated farmland patches. In particular, the leapfrogging built-up lands in the exurban portions of the urban planning area, especially those dispersing throughout where formerly farmland covered, have noticeably reduced farmland.

Land use changes in the non-urban planning area are always administered by a permit system of rural land use control system named rural land contracting regulation, but this system was not guided by the land use planning. Due to the shortage and high price of land in the urban planning area, built-up land sprawl has also emerged in the non-urban planning area. There was considerable built-up land sprawl in the non-urban planning area between 1994 and 2003. It was intensified by the lack of supervision on land use in the non-urban planning area. Nearly 29% of farmland was lost in the non-urban planning area, and the total area of built-up land doubled during the ten years.

Land use exhibited different spatial characteristics in the two planning area. Statistical results showed that, for the period 1994–2003, built-up land within urban planning area represented 60.7% of the total new development in Hang-Jia-Hu region. The remaining 39.3% of built-up land was distributed across the non-urban planning area. The rapid built-up land sprawl was the main contributor to farmland conversion in this region. Statistics showed that 28.5% farmland had been lost in the ten years, 63.6% of which was concentrated in the non-urban planning area. Calculating the rate of farmland conversion for both planning areas, we found that the rate of the non-urban planning area was higher than that of the urban planning area. The built-up land sprawl in the non-urban planning area was responsible for the higher rate of farmland conversion. Besides, the NP and LPI of farmland in the non-urban planning area decreased, which may be linked with the land consolidation practices in coastal rural areas. During land consolidation, small plots were aggregated into large patches and they were regulated into more regular forms.

To better understand the influences of different land use spatial control schemes on farmland conversion and urban development, we further made a comparison of the statistical results of landscape metric analysis. Generally, built-up land in the urban planning area showed a dual trend of fragmentation and aggregation, while farmland presented a single trend of fragmentation. Different from the urban planning area, built-up land in the non-urban planning area presented a trend of aggregation, while farmland exhibited a trend of fragmentation. Specially, farmland in the non-urban planning area was more fragmented than that found in the urban planning area. There were increases of PD and SHAPE for both planning areas, suggesting that farmland fragmentation was intensified. Besides, the fragmentation of built-up land in the non-urban planning area was more significant than in the urban planning area. Built-up land sprawl in the form of continuous development in the urban planning area led to farmland conversion through spreading into the adjacent farmland. In the non-urban planning area, built-up land sprawl in the form of leapfrogging development resulted in farmland areal declines and fragmentation. Combined with the Moran’s I values, it could be inferred that the leapfrogging built-up land sprawl further intensified the fragmentation of built-up land in the non-urban planning area.

This study evidenced that farmland conversion and urban development could present different spatial characteristics under different land use spatial control schemes. The issue of farmland conversion to built-up land was more serious in the non-urban planning area. The high rate of farmland conversion was largely a reflection of the poor legal system and law enforcement measures in rural China. A guiding land use planning should be proposed for the non-urban planning area, and it should be integrated with the land use planning for the urban planning area. It should be mentioned that the interactions among urban development, farmland conversion and land policy schemes are very complex [22,38,39], since socioeconomic factors and individual behaviors are involved. Consequently, we should not merely rely on standard spatial planning to cope with the conflict between farmland protection and urban development. Future studies should incorporate more influential factors to discuss the links among urban development, farmland conversion and spatial planning.
